# Knowledge, attitude, and practice of dentists regarding periodontal tissue health in Birjand, Northeast Iran

**DOI:** 10.34172/japid.2021.006

**Published:** 2021-05-16

**Authors:** Marzieh Mohammadi-Moghaddam, Mohaddese Zebarjadi, Freshteh Osmani

**Affiliations:** ^1^Department of Periodontics, School of Dentistry, Birjand University of Medical Sciences Birjand, Iran; ^2^Dentistry Clinical Research Development Center, Birjand University of Medical Sciences, Birjand, Iran; ^3^Student Research Committee, Birjand University of Medical Sciences, Birjand, Iran; ^4^Infectious Diseases Research Center, Birjand University of Medical Sciences, Birjand, Iran; ^5^Department of Epidemiology and Biostatistics, Faculty of Health, Birjand University of Medical Sciences, Birjand, Iran

**Keywords:** Attitude, Dentists, Knowledge, Periodontal tissue, Practice

## Abstract

**Background:**

Determining what dentists know and believe about periodontal tissue properties is important to establish prevention practices. The present study aimed to investigate the knowledge, attitude, and practice of general dental practitioners about the properties of periodontal tissue around retainable teeth in Birjand, Northeast Iran.

**Methods:**

The knowledge, practice, and attitude of 91 dentists about periodontal tissue properties around retainable teeth were assessed by a validated researcher-made questionnaire.

**Results:**

The results showed that the mean score of dentists’ attitude, knowledge, and practice were 70.7, 88.2, and 77, respectively. The mean score of the attitude of male dentists was higher than females significantly (*P*=0.014).

**Conclusion:**

It is highly recommended that continuing courses should be held to improve their knowledge.

## Introduction

Periodontal health is essential for the long-term preservation and restoration of teeth. Periodontal diseases, including gingivitis and periodontitis, are prevalent worldwide. In both developed and developing countries, severe periodontitis is estimated to affect 5%–20% of adults.^1^

Different clinical methods were expanded to hinder downward epithelial migration and promote periodontal tissue regeneration to regenerate healthy periodontal tissues.^2^ Guided tissue regeneration and guided bone regeneration create a three-dimensional space between the defect and root/bone surface to regenerate periodontal ligament (PDL) and bone tissue. Although these techniques are useful in current dental treatment, a long-term stable clinical outcome has not been achieved. For reasons such as aesthetic considerations, the spread of caries or fractures below the gingival margins, perforations caused by endodontic or restorative treatments, etc., increasing the length of the clinical crown is prescribed.^3,4^

Increasing the clinical crown length can be achieved in two ways: surgery to increase the length of the clinical crown and extrusion of teeth with orthodontic forces.^5^ The main actions of the periodontium consist of protecting teeth and blood vessels from injury by mechanical forces and attachment of the teeth to bone. Surgery is usually preferred if two or more teeth need to be increased clinically. This operation is performed by removing the gingiva and bone in the area of the tooth below the gingival margin so that the healthy part of the tooth is completely outside the gingivae and bones.^6^ The present study aimed to assess the knowledge, attitude, and practice of general dental practitioners concerning periodontium properties in Birjand, Iran, in 2020.

## Methods

This cross-sectional analytical study was carried out on 91 general dental practitioners in Birjand, Northeast Iran in 2020. A questionnaire was submitted to the dentists. This questionnaire consisted of demographic characteristics and questions about knowledge, attitude, and practice regarding the characteristics of the periodontal tissue around the retainable teeth. The validity of the questionnaire was assessed by nine relevant experts, and questions with a CVR of <0.75 were removed. Reliability was confirmed by Cronbach’s alpha test (α=0.81 for knowledge, α=0.72 for attitude, and α=0.79 for practice). Each question had a Likert scale score from 1 to 5 in the knowledge and attitude section, scored the highest according to the correctness of the answer.

The practice section consisted of nine 3-choice questions to show how dental practitioners adhered to the principles and, if necessary, whether they referred the patient to a specialist. The participants were given a score between 1 and 3 according to the correct answer. To determine the levels of knowledge, it was divided into poor, moderate, and good. To determine the levels of attitude, it was classified into poor, moderate, and good.

Quantitative data were reported as mean (±SD), and qualitative data were shown as frequency and percentage.

## Results

Thirty-nine subjects (44.8%) were female, and 48 (55.2%) were male; 44.8% of dentists had less than ten years of experience, 33.3% had 10‒20 years of experience, and 21.8% had more than 20 years of experience (Table 1), 50.6% in the private practice, 39.1% in the clinic, and 10.3% in both.

**Table 1:** Demographic characteristics of dentists participating in the study

**Variable**	**Status**	**Frequency (n)**	**Percent**
Gender	Male	48	55.2
	Female	39	44.8
Work Experience	>10	39	44.8
	10-20	29	33.3
	<20	19	21.8
Location	Private practice	44	50.6
	Clinic	34	39.1
	Both	9	10.3

The knowledge levels of dentists were 62.1% and 37.9% as moderate and good, respectively. All the participants had good attitudes about the characteristics of periodontal tissues around retainable teeth. Regarding dentists’ practice levels, 5.7% had moderate, and 94.3% had good practice, and none had poor practice (Table 2).

**Table 2:** The level of knowledge, attitude, and practice among participating dentists

**Level**	**Knowledge**		**Attitude**		**Practice**	
	**Number**	**Percent**	**Number**	**Percent**	**Number**	**Percent**
Poor	0	0	0	0	0	0
Medium	54	62.1	0	0	5	5.7
Good	33	37.9	87	100	82	94.3
Total	87	100	87	100	87	100

The total mean score of attitude was 12.49±3.53. Male dentists had a significantly higher score than female dentists (*P*= 0.014; Table 3). There was a significant difference in knowledge based on work experience so that dentists with a history of more than 20 years had the lowest level of knowledge.

**Table 3:** Comparison of mean scores for knowledge, attitude, and practice regarding periodontal tissue characteristics among participating dentists by gender

**Scale**	**Gender**	**N**	**Mean ± SD**	***P*** ** value**
Knowledge	Male	48	11.81 ± 1.77	0.264
	Female	39	12.28 ± 2.12	
Attitude	Male	48	49.22 ± 2.52	0.014
	Female	39	47.66 ± 3.27	
Practice	Male	48	23.20 ± 2.10	0.159
	Female	39	22.46 ± 2.79	

*P* value: Independent t-test.

## Discussion

Clinical decision-making has been a very important topic in the last decade. Unfortunately, diagnosis and treatment planning are problematic skills that have not been well emphasized in the education of dental students in the past.^1^

This study aimed to assess the level of knowledge, attitude, and practice toward the characteristics of periodontal tissues around retainable teeth among dentists.

The results of this study showed that participants’ knowledge was slightly lower than the average. Participants who worked in governmental clinics were more knowledgeable than those in private clinics.

In this study, most participants had a moderate level of knowledge. In a study on dental students’ knowledge about oral health and periodontal health, many participants were aware of this issue.^7^

Our results showed that all the participants had a positive general and personal attitude towards characteristics of periodontal tissues around retainable teeth. Generally, this finding was similar to a study in South Africa,^8^ showing that the majority had a positive attitude. However, it was difficult to make item-by-item comparisons due to the discrepancy in designed questions and assessment methods. In the present study, 37.9% of dentists had good knowledge, and 94.3% showed good performance. Lower knowledge and higher performance in the present study than other studies might be attributed to differences in the study sample.

## Conclusion

According to the results of this study, more attention should be directed towards policy-making for educational programs. Moreover, due to the importance of periodontal health, the incorporation of programs that can promote closer interactions between oral health and the general health can enhance the practice of dentists.

## Authors’ contributions

MM and MZ designed the experiments. MZ collected data. FO performed the statistical analyses and wrote the results section. FO and MM interpreted the results. MZ wrote the initial manuscript. FO critically reviewed and modified the manuscript. All authors have read and approved the final manuscript.

## Availability of data

The datasets used and/or analyzed during the current study are available from the corresponding author on reasonable request.

## Ethics approval

This article was extracted from a dissertation approved by the Ethics and Scientific Committee of Birjand University of Medical Sciences, Iran, under the code the code IR.BUMS.REC.1399.033.

## Competing interests

The authors declare that they have no competing interests.

## References

[1] Hamissi J, Poursamimi J, Modarres A (2011). Knowledge, attitude, and practice of general dental practitioners on aggressive periodontitis in Qazvin. J Qazvin Univ Med Sci.

[2] de Waal H, Castellucci G (1994). The importance of restorative margin placement to the biologic width and periodontal health Part II. Int J Periodontics Restorative Dent.

[3] Patil S, Thakur R, K M, Paul ST, Gadicherla P (2013). Oral health coalition: knowledge, attitude, practice behaviours among gynaecologists and dental practitioners. J Int Oral Health.

[4] Osmani F (2020). Statistical ambiguities in epidemics of coronavirus disease 2019 (COVID-19). Acta Med Iran.

[5] Boggess KA, Urlaub DM, Massey KE, Moos MK, Matheson MB, Lorenz C (2010). Oral hygiene practices and dental service utilization among pregnant women. J Am Dent Assoc.

[6] Atarbashi Moghadam F, Haerian Ardakani A, Rashidi Meybodi F, Khabazian A (2013). Evaluation of periodontal health knowledge, attitude and oral hygiene practice of pregnant women in Yazd in 2011. J Periodontol Implant Dent.

[7] Al-Khabbaz AK, Al-Shammari KF, Al-Saleh NA (2011). Knowledge about the association between periodontal diseases and diabetes mellitus: contrasting dentists and physicians. J Periodontol.

[8] Grossman ES, Naidoo S (2009). Final-year South African dental student attitudes toward a research component in the curriculum. J Dent Educ.

